# Bacterial generalists and fungal specialists play important roles in maintaining community stability and regulating microbial diversity of the algae-associated microbiome throughout the growth cycle of *Alexandrium pacificum*

**DOI:** 10.1128/aem.01359-25

**Published:** 2025-09-22

**Authors:** Yanlu Qiao, Lingzhe Wang, Shuo Wang, Shijie Li, Feng Wang, Bo Wang, Siheng Lin, Yuyang Liu

**Affiliations:** 1 College of Safety and Environmental Engineering, Shandong University of Science and Technology, Qingdao, Shandong, China; 2 Department of Biological Sciences and Biotechnology, Minnan Normal University 58299, Zhangzhou, Fujian, China; 3 Institute of Yellow River Delta Earth Surface Processes and Ecological Integrity, Shandong University of Science and Technology, Qingdao, Shandong, China; Georgia Institute of Technology, Atlanta, Georgia, USA

**Keywords:** niche, ecology, evolution, microbes, harmful algal bloom

## Abstract

The algae-associated microbiome represents a consortium that has been chronically domesticated by specific algae, maintaining a close relationship with the host population. Although the microbes associated with dinoflagellates have garnered significant attention in recent years, the interactions and contributions of microbial generalists and specialists through the growth cycle of a bloom-forming dinoflagellate remain largely unexplored. Herein, the ecological and evolutionary characteristics of free-living generalists and specialists within bacterial and fungal communities were investigated during the growth process of *Alexandrium pacificum* cultured in the laboratory for years without antibiotic treatment. From an ecological perspective, bacterial generalists and fungal specialists dominated the community, indicating different microbial niche patterns between the bacterial and fungal consortia. Furthermore, microbial specialists were more susceptible to disturbance from algal proliferation, as evidenced by greater community dissimilarity and determinacy-dominated assembly processes. Compared with their counterparts, the molecular networks of bacterial generalists and fungal specialists were more complex and robust, suggesting that they significantly contributed to resistance to environmental stress and functional maintenance. Evolutionarily speaking, bacterial generalists and fungal specialists showed much higher diversification potential, and others featured higher extinction rates. Despite these differences, a continuous transition from the former two to their counterparts was observed, alleviating the “Matthew effect” in the biological world for ecological trade-offs. Collectively, these findings emphasize that bacterial generalists and fungal specialists play important roles in maintaining community stability and regulating microbial diversity during the growth process, which expands the current understanding of the maintenance mechanisms of the diversity and community of phytoplankton-associated microbes in the face of disturbance from bloom-forming dinoflagellates.

**IMPORTANCE:**

Like the microbes residing in the rhizosphere and human gut, bacteria that coexist chronically with microalgae exemplify a relationship, forming potentially intimate partnerships with the host. However, studies on the ecological significance of algae-associated microbiomes with different niches under the interference of bloom-forming species are still lacking. This work investigated the ecological interactions and contributions of generalists and specialists within algae-associated bacterial and fungal communities across the growth cycle of *Alexandrium pacificum* for the first time. These results increase the understanding of the microbial ecology of algae-associated microbes in the context of interference from the proliferation of harmful algal bloom species.

## INTRODUCTION

Microorganisms are almost ubiquitous around the world and play pivotal roles in diverse ecosystems (1). They are characterized by the presence of niche generalists and niche specialists (2–4), depending on their diversity, occurrence, and niche breadth. In general, generalist species exhibit wide environmental tolerances, whereas specialists inhabit a narrow range of habitats with specific environmental fitness requirements (5, 6). Both generalists and specialists play a fundamental role in maintaining the resilience and functioning of ecosystems. Their interactions, competitive dynamics, and coexistence profoundly shape the ecological landscape, significantly influencing overall species richness and community (7). Unraveling the processes and mechanisms that drive the dynamic patterns of microorganisms is essential in microbial ecology (8, 9) to better understand the responses of ecosystems to environmental change and deduce their future states. Thus, in addition to the whole community, considerable efforts have been made to elucidate the ecological and evolutionary processes of microbial generalists and specialists through investigations of their distribution patterns, community assembly (deterministic and stochastic processes), co-occurrence networks, speciation, and extinction using DNA metabarcoding. For example, in the natural environment, a high speciation rate of niche specialists has been reported across temperature gradients in hot springs, alleviating the negative effect of environmental filtering on microbial diversity (2). With respect to bioremediation systems, bacterial specialists play a pivotal role in preserving system stability during the oil removal process under deep-sea conditions (10). By surveying niche microorganisms, these reports provide a deep understanding of the mechanisms underlying microbial community dynamics and biodiversity maintenance, microbe-to-microbe interactions, and changing trends in ecosystem functions in the context of environmental changes and anthropogenic influences.

Harmful algal blooms (HABs) have emerged as significant natural disasters, garnering considerable attention in recent decades (11), posing a threat to public health and aquatic ecosystems (12). Dinoflagellates, a phylum of unicellular microalgae, serve as crucial primary producers within marine ecosystems, functioning as either free-living plankton (auto, hetero, or mixotrophic) or endosymbionts within coral reefs, and many of them are known as HAB-causing species (13). At present, microbes are thought to be closely related to dinoflagellate HAB processes (14–16). Among them, a specific assemblage of microbes (including those with free-living and attached lifestyles) associated with HAB species exhibits long-term coexistence and maintains a close relationship with host algae (17–20), for instance, their effects on host function and overall fitness (17, 21, 22). Based on the study of Kim et al. (23), these microorganisms can be referred to as the “algae-associated microbiome.” These studies help understand the specific relationships between a microalgal host and long-term microbes, contributing to a better understanding of complex algae‒microbe interactions. Nonetheless, precisely characterizing the microbial consortia that coexist with phytoplankton in aquatic habitats poses a considerable challenge, primarily because of the unicellular traits of most microalgae and because they live in constantly shifting and dynamic environments (24). Perhaps long-term coculture of dinoflagellates and microbes allows us to obtain stable microbial communities, and such bioconsortia could be deemed algae-associated microbes (17, 18, 23). Hence, most explorations on their composition, diversity, and function have been conducted mainly in laboratory-reared dinoflagellates cultured for months to years (17, 25, 26), improving our understanding of algal‒microbe interactions and providing important links between laboratory and field data. With respect to microbial generalists and specialists in the phycosphere, few investigations have been conducted. For instance, the distribution patterns of host-specific and generalist species have been described among several macroalgal genera (27). Recently, the dynamic changes and functional characteristics of generalists and specialists have been revealed in 15 phytoplankton isolates (including diatoms and green algae) over long-term culture (19). Undoubtedly, these findings provide important insight into how the phytoplankton microbial community and related functions are shaped by microbial categories with different ecological strategies. However, to the best of our knowledge, how generalists and specialists interact as well as what contributions they provide to microbial community establishment and diversity maintenance during algal growth remains largely unexplored, particularly those in HAB-forming dinoflagellate species. This knowledge gap impedes a deeper understanding of the ecological significance of the two niche microbes in the involved process.

Among HAB-causing species, *Alexandrium pacificum* (*A. tamarense* Group IV) is widely distributed worldwide and produces paralytic shellfish toxins (28). Taking this species as an example, in the present study, a growth cycle of *A. pacificum* (long-term indoor cultivation without antibiotic treatment), including the initial, plateau, and last stages, was constructed. Given that free-living microbes are widely distributed and are considered fundamental components of marine ecosystems, with their significant contributions to biomass, decomposition, and primary production (29, 30), this study focused on the ecological (including composition, diversity, interaction patterns, and community assembly) and evolutionary (including species speciation, extinction, and transition rate) characteristics of free-living generalists and specialists within algae-associated bacterial and fungal communities during the growth process. These results expand the current understanding of microbial ecology under disturbance from the proliferation of HAB species, particularly in terms of interspecies interactions, community assembly, and biodiversity maintenance of algae-associated microorganisms with different niches, and thus provide deeper insight into the microbial response to HABs.

## MATERIALS AND METHODS

### Algal cultures and cultivation

The *A. pacificum* strain NJD-1 utilized in the current study was isolated from seawater off the coast of Zhejiang, China, without any creatures other than microbes (barely detectable to the naked eye) that were carried by micropipette. The culture was cultivated for years without antibiotic treatment in sterile natural seawater supplemented with f/2-Si medium (31) under controlled conditions of 21 ± 1°C, ~100 µmol quanta·m^−2^·s^−1^, and a photoperiod of 12:12 h light:dark, provided by white fluorescent lights (Ningbo Jiangnan Instrument Factory, China). Following enrichment, 500 mL solutions containing 100 mL of algal culture and 400 mL of culture medium were transferred into eight sterile 1 L conical flasks in triplicate (Fig. S1). For sampling purposes, triplicate 1 mL samples were collected from each flask at each designated time point (*N* = 8), and subsequent cell counts were performed using Lugol’s reagent at a final concentration of 2%. The algal cell density was measured every 2 days for the initial 14 days and then at 4-day intervals for the subsequent 40 days. Based on the density, three distinct growth stages were defined as follows (Fig. S1): AP1 and AP2 (initial stage); AP3, AP4, and AP5 (plateau stage); and AP6, AP7, and AP8 (last stage). This method has been used in another study on the construction of simulated dinoflagellate blooms in the laboratory (25).

### DNA extraction, sequencing, and data processing

Each sample at the same time point underwent filtration using 3 µm and subsequently 0.22 µm polycarbonate membranes (Millipore, USA) to capture free-living microbial cells. The membranes were then stored at −80°C until further extraction. Total DNA was extracted using the Fast DNA SPIN Kit (MP Biomedicals, Santa Ana, CA, USA) according to the manufacturer’s protocol. The concentration and purity of the extracted DNA were assessed using a NanoDrop 2000 UV-spectrophotometer (Thermo Scientific, Wilmington, USA). In bacteria, the V3–V4 region of the 16S rRNA gene was amplified via PCR with the primers 338F (5′-ACTCCTACGGGAGGCAGCAG) and 806R (5′-GGACTACHVGGGTWTCTAAT). For fungi, the ITS region was amplified using a thermocycler PCR system with the primers ITS1F (5′-GCATCGATGAAGAACGCAGC) and ITS2R (5′-TCCTCCGCTTATTGATATGC). The resulting amplicons were sequenced on the Illumina MiSeq PE300 platform (Illumina, San Diego, USA) at Majorbio Bio-Pharm Technology Co., Ltd. (Shanghai, China). The obtained data were subjected to quality control using Trimmomatic software (version 0.33) and Cutadapt (version 1.9.1) to obtain clean data. Chimeric sequences were subsequently removed utilizing UCHIME. Operational taxonomic units (OTUs) were then generated at a 97% classification level using VSEARCH. OTUs with read counts less than two that may represent sequencing errors were removed for downstream analyses. The taxonomy of the OTUs was categorized using the SLIVA132 and NT databases.

### Identification of niche specialists and niche generalists

Several studies on microbial specialists and generalists have been reported in closed or open environments (2, 3, 10, 32). The concept of “niche breadth,” originally proposed by Levins (33), has been utilized to distinguish different degrees of species specialization (10, 34). Based on other literature reports (10, 34, 35), to identify specialists and generalists, B values were calculated using the formula described by Levins (33):


$${\text{B}}_{j}\text{=}\frac{\text{1}}{\sum_{i\text{=1}}^{N}P_{ij}^{\text{2}}}$$

*B*_*j*_ represents the niche breadth, and *P*_*ij*_ denotes the relative abundance of species *j* in a specific habitat *i*, corresponding to different growth stages. The classification of generalist and specialist taxa was conducted at the OTU level. OTUs that exhibited a broad presence across multiple samples with a more even distribution were assigned higher *B* values, indicating their generalist nature. Conversely, OTUs with lower *B* values were categorized as specialists, reflecting their more restricted distribution (34). To avoid potential misinterpretation as specialists, OTUs with a mean relative abundance of less than 2 × 10^−5^ were excluded from the analysis, following the approach outlined by Pandit et al. (36). The niche breadth for bacteria ranged from 1 to 7.6 (with a theoretical maximum of 8), whereas that of fungi fluctuated from 1 to 5.1 (with an approximate maximum of 5). Generalists were defined operationally as OTUs with *B* values exceeding 4 (for bacteria) and 2.5 (for fungi), with generalist OTUs occurring in more than four samples (*N* = 8). The thresholds were generally selected based on the median of B values, which has been widely used in other studies (10, 34). By contrast, bacterial and fungal specialists were defined as OTUs whose *B* values were less than 1.5, which is close to the lower limit of 1 for B values (10, 34). These specialist OTUs were primarily observed in just one or two samples.

### Alpha and beta diversity

The assessment of α diversity, quantified by the Shannon (biodiversity) and Chao 1 (richness) indices, was conducted using Quantitative Insights Into Microbial Ecology (QIIME) software (37). Significant differences were determined using one-way analysis of variance (ANOVA) followed by the least significant difference (LSD) test. For β-diversity, principal coordinate analysis (PCoA) based on OTUs was employed, utilizing abund-jaccard distances in R programming. The ANOSIM method, with 999 permutations, was employed to evaluate the statistical significance of community grouping using QIIME software.

### Co-occurrence network of the microbial community

A molecular ecological network was used to analyze the co-occurrence patterns between generalists and specialists, revealing complex microbial interactions that are essential for maintaining community diversity and ecosystem functions (38, 39). The construction of the network was executed by integrating the “igraph,” “psych,” and “WGCNA” packages within the R programming environment. To ensure robust correlations, Spearman’s correlation coefficients (r > 0.6) with corresponding *P* values < 0.05 were used to establish connections in the networks (40). *P* values were adjusted using the Benjamini and Hochberg procedure (41). Furthermore, to explore the potential contributions of generalists and specialists in molding the microbial community during cell growth, induced subgraphs of these taxa were extracted separately. The topological properties of the whole network (all microbes) and subnetworks (bacteria or fungi) were assessed using the “igraph” package to characterize the network structure. Network visualization was achieved through the interactive platform Gephi (version 0.9.2). As mentioned above, the significance test was performed using ANOVA. Furthermore, to evaluate the natural connectivity of the network, an “attack” simulation on random nodes within the static subnetworks of generalists and specialists was conducted. Notably, a robustness test was implemented as a powerful tool to assess network stability, specifically measuring changes in natural connectivity upon node removal. Moreover, to assess the natural connectivity of the network, a simulation was conducted in which random nodes within the static subnetworks of generalists and specialists were attacked. Notably, this robustness test served as a valuable tool to evaluate the stability of the network by measuring alterations in natural connectivity following the removal of nodes (10).

### Community assembly process and phylogenetic diversity

To reveal the relative importance of deterministic and stochastic processes in community assembly, we utilized normalized stochasticity ratios (NSTs) through the “tNST” function within the “NST” package in R (42). NST is a metric that quantifies ecological stochasticity in systems, providing a statistical framework to evaluate whether communities exhibit greater or lower similarity than the null expectation driven by deterministic factors (43). NST values range from 0 to 1, where a value of 0 implies primarily deterministic-driven community assembly, and a value of 1 indicates the complete predominance of stochasticity in the assembly process. To differentiate between predominantly deterministic (NST <0.5) and more stochastic (NST >0.5) assembly processes, a threshold of 0.5 was established. To assess microbial phylogenetic diversity while controlling for species richness effects, the mean nearest-taxon distance (MNTD) metric was employed. The metric, calculated using the “mntd” and “ses.mntd” functions in the “picante” R package, quantifies the phylogenetic distinctiveness at the terminal branches of the phylogenetic tree (10). MNTD measures the average branch length between each OTU and its closest phylogenetic relative within a sample through 999 random resamplings from a source pool based on a null model (10). A lower MNTD value indicates closer phylogenetic affinity among the terminal branches. Differences in these parameters between specialists and generalists were tested by ANOVA.

### Evolutionary trends of specialists and generalists inferred by BISSE models

The binary-state speciation and extinction (BiSSE) model is a phylogenetic tree-based approach used to assess the binary evolutionary characteristics of species. It specifically examines speciation (λ), extinction (μ), and state transition (t) rates using long or short sequencing reads (2, 10). In this study, the BiSSE model was applied to investigate the evolutionary traits of specialists and generalists, following established methodologies employed in previous research conducted in both open and closed environments over short or long experimental durations (3, 10, 32). The BiSSE model was implemented using the “diversitree” R package in R programming (2). It differentiates between specialists and generalists as discrete evolutionary states and calculates their respective speciation and extinction rates, allowing for the estimation of transitions between these ecological states. The BiSSE model determines the optimal rate parameters that maximize the model’s fit to the observed data, with the phylogenetic tree serving as a crucial input. Consistent with other studies (2, 10), a unified phylogenetic tree was constructed using FastTree based on the sequencing data in this study. This model consists of two steps. Step 1 establishes an initial simulation point using the “starting.point.bisse” function. The speciation and extinction rates of the habitat generalists and specialists were constrained to be identical. In Step 2, all rates are permitted to vary, and the maximum likelihood method is employed to compute the evolutionary rate parameters using the “find.mle” function (10, 44). To ensure the robustness and stability of the final parameter estimates, a Markov chain Monte Carlo (MCMC) simulation with 1,000 and 5,000 permutations was conducted (10), and similar results were obtained between the two types of simulations.

## RESULTS

### General analysis of 16S rRNA gene sequencing data

The rarefaction curve of the Shannon index, which was generated based on the OTU data, tended to flatten (Fig. S2), and the Good’s coverage values for both the bacterial and fungal sequencing data exceeded 99%. These findings indicate that the sequencing depth was sufficient and that the sequencing data captured the majority of bacterial and fungal species in the samples. Following quality filtering, a total of 619,543 and 535,204 high-quality sequences were obtained for the bacterial and fungal communities, respectively, using PCR primers targeting the partial 16S and ITS rDNA regions. After OTU classification and rarefaction, 270 (35.5%) and 62 (3.4%) OTUs were identified as strict bacterial generalists and specialists, representing 80.3% and 0.5% of the total bacterial sequences, respectively. A total of 143 generalist OTUs (9.5%) and 1,147 specialist OTUs (75.9%) were identified in the fungal community, with relative abundances of 11.8% and 84.0%, respectively. The niche breadths of both bacterial and fungal generalists were clearly wider than those of specialists (*P* < 0.001; Fig. S3).

### Diversity, composition, and community assembly of microbial specialists and generalists

**Fig 1:**
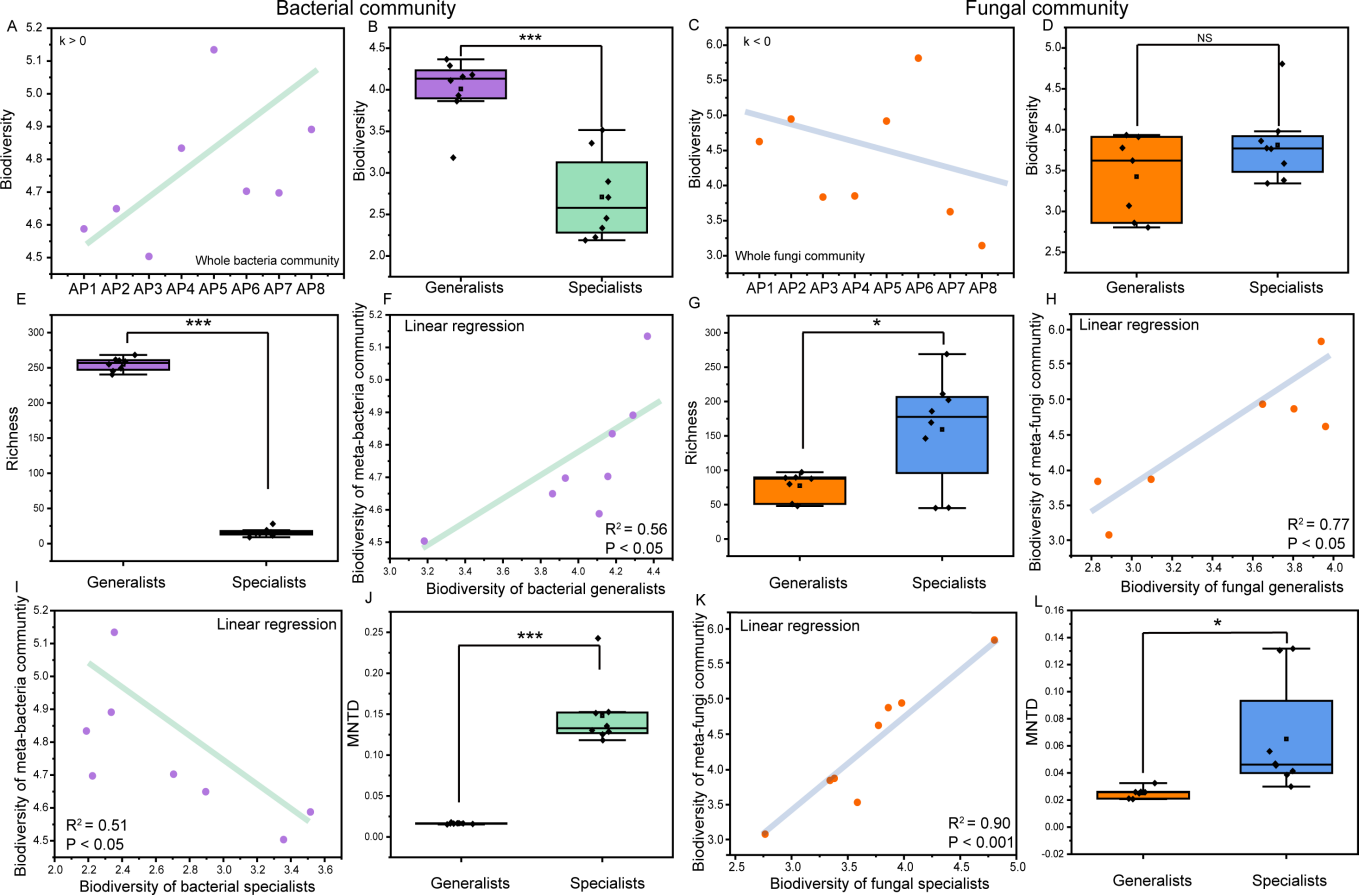
Diversity patterns for microbial generalists and specialists in the growth process. (**A, C**) Changing trend of the biodiversity of the whole bacterial and fungal community. Comparisons of diversity (**B, D**) and richness (**E, G**) between microbial generalists and specialists. (**F, H, I, K**) Linear correlations between the diversity of niche microbial taxa and the whole community. (**J, L**) Variations in the community mean nearest-taxon distance between microbial generalists and specialists. *P < 0.05; **P < 0.01; ***P < 0.001.

**Fig 2:**
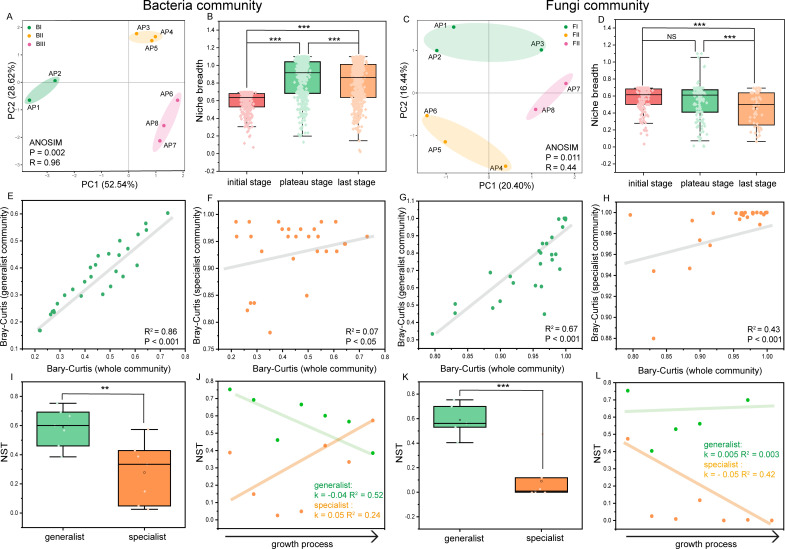
Dynamics of bacterial and fungal communities along the process. (**A–D**) PCoA based on OTUs and niche breadth among growth stages. (**E–H**) The relationship between generalists’ and specialists’ community structure with the whole community structure. (**I–L**) Comparison of NSTs between generalists and specialist communities and the value variation along the process. *P < 0.05; **P < 0.01; ***P < 0.001. NS indicates not significant.

The Shannon indices (biodiversity) of the microbial community ranged from 4.50 to 5.13 (for bacteria) and from 3.14 to 5.81 (for fungi) (Fig. 1), with no significant difference between the two communities (*P* > 0.05), but the mean value was relatively high (4.75 ± 0.20) in the former (Fig. 1A and C). By contrast, a significantly greater richness based on the Chao 1 index was detected for bacterial taxa (*P* < 0.01). With increasing algae growth, bacterial diversity tended to increase, whereas the opposite trend occurred in the fungal community (Fig. 1A and C). With respect to the subcommunity, the richness and diversity of bacterial generalists and specialists significantly differed during algal proliferation, with a significantly greater number of generalists (Fig. 1B and E; *P* < 0.001). By contrast, fungal specialists exhibited distinctly higher richness (*P* < 0.05) and greater diversity (*P* > 0.05) compared with those of generalists (Fig. 1D and 2G). In addition, there was a positive relationship between the biodiversity of the bacterial consortium and generalist (*P* < 0.05; Fig. 1F), whereas a contrasting phenomenon was observed between the bacterial consortium and generalists (*P* < 0.05; Fig. 1I). The levels of diversity of both fungal specialists and generalists were positively correlated with that of the whole community (*P* < 0.05; Fig. 2H and K). With respect to phylogenetic diversity, compared with generalists, both bacterial and fungal specialists had higher MNTD values (*P* < 0.05; Fig. 1J and L), indicating greater evolutionary differences among the former.

In both taxa, Bray‒Curtis pairwise distance PCoA at the OTU level revealed clear community heterogeneity during algae growth (R = 0.96, *P* = 0.002 for bacterial taxa; R = 0.44, *P* = 0.011 for fungal taxa) (Fig. 2A and C). From the pre- to post-stage, the community niche breadth significantly increased and decreased, respectively, for the bacterial and fungal taxa (*P* < 0.001; Fig. 2B and D), indicating that more resources were available to the former. In addition, the microbial specialists exhibited greater dissimilarity between any paired samples, with little overlap with the overall community (Fig. 2F and H). By contrast, the abundance of microbial generalists was strongly correlated with that of the whole community (Fig. 2E and G). For the community assembly process, overall, the NST values of generalists calculated with the abundance-based Ružička metric were mostly greater than 0.5, with averages of 0.59 ± 0.13 (bacteria) and 0.59 ± 0.14 (fungi), indicating a more important role of stochastic processes (Fig. 2I and K). By contrast, determinacy factors dominated the specialist subcommunity assembly because most NSTs were less than 0.5 for both bacterial (average value of 0.28) and fungal (average value of 0.08) taxa (Fig. 2I through L), indicating that the microbial specialist community is more susceptible to algal proliferation. Interestingly, during algae growth, the determinacy-dominated role gradually increased in bacterial generalist assembly, and an increased stochastic effect was observed in specialist taxa. However, those in the fungal community exhibited the opposite phenomenon (Fig. 1J and L).

Furthermore, 31 and 19 bacterial genera were identified as generalists and specialists, respectively. They belonged to Proteobacteria and Bacteroidota. At the genus level, 5 generalists and 83 specialists were found in the fungal community, which were classified mainly into Ascomycota and Basidiomycota. Changes in the relative abundances of the top 20 genera of bacteria and fungi across algae growth are shown in Fig. S4. For bacteria, the principal generalists and specialists were unclassified genera of Rhodobacteraceae, *Marixanthomonas*, and *Algoriphagus*, which accounted for approximately 83% and 53% of the total abundance in their own communities, respectively (Table S1). In addition, rare bacterial taxa, such as *Tateyamaria*, *Actibacterium,* and *Nioella,* were observed only as generalists, whereas species such as *Fabibacter* and *Sulfitobacter* tended to be the sole specialists (Table S1). With respect to fungi, *Apiotrichum*, *Meyerozyma,* and *Penicillium* were the dominant generalist taxa, accounting for approximately 84% of the total reads (Table S1). Several genera, such as *Alternaria*, *Aspergillus*, and *Papiliotrema,* were the most abundant specialist species (Table S1).

### Molecular ecological networks of generalist and specialist taxa

**Fig 3:**
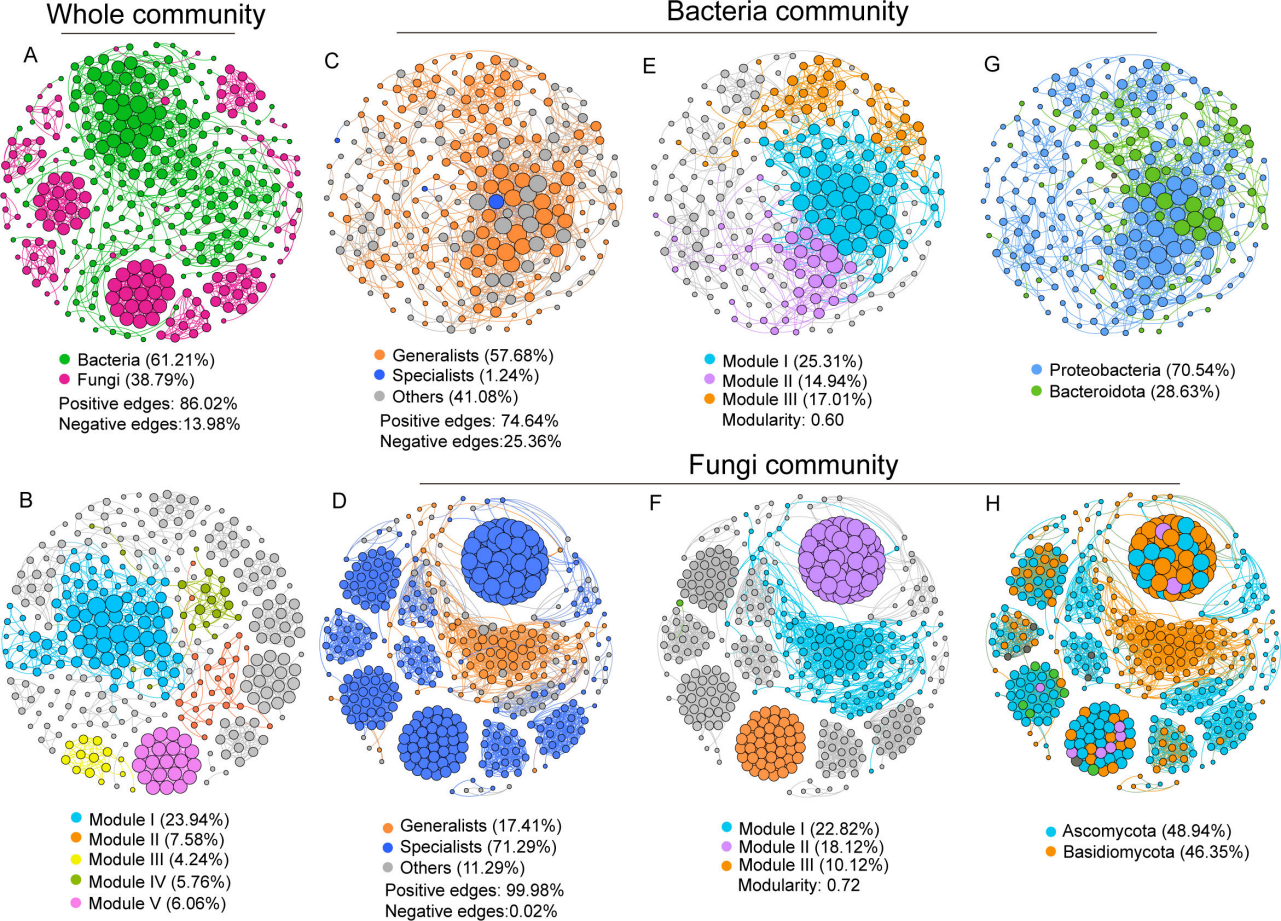
Co-occurrence networks of whole- (including bacteria and fungi) and sub-community based on OTUs. The size of dots is proportional to node degree. Colored co-occurrence networks show the distribution of modules, phylum-level taxa, and niche microbes within the communities. (**A, B**) Network of the whole bacterial and fungal community. (**C, D**) Distribution patterns of generalists and specialists within bacterial (**C**) and fungal (**D**) networks, respectively. Distribution of modules (**E, F**) and microbial species (**G, H**) in networks.

**Fig 4:**
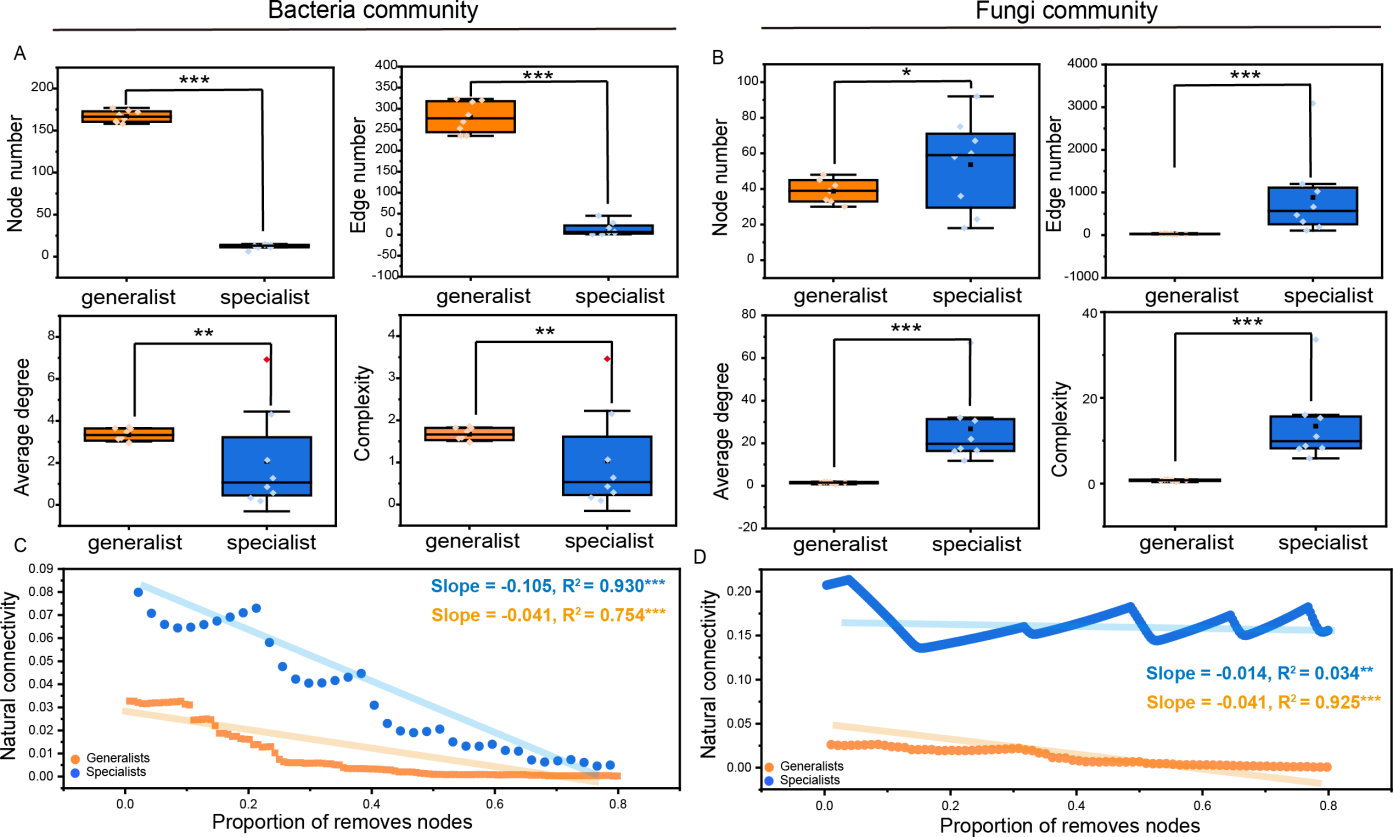
Comparisons of topological properties and robustness analysis between generalist and specialist networks. (**A, B**) Multiple network properties, including nodes, edges, average degree, and complexity. Red dots represent outliers that were not used in statistical tests. (**C, D**) Robustness analysis conducted as the relationships between the natural connectivity of generalists and specialists and the proportion of removed nodes, such that larger shifts upon the same proportion indicate that there is less stability within networks. *P < 0.05; **P < 0.01; ***P < 0.001.

To investigate potential ecological interactions among species, based on Spearman correlations among OTUs, we constructed separate co-occurrence networks for the whole community (containing bacteria and fungi) (Fig. 3A and B) and the subcommunity (Fig. 3C through H) during algae growth. In the whole network, the microbial interactions were relatively stable, as the network size displayed slight variations among the growth stages, ranging from 579 to 784. Bacterial and fungal taxa accounted for 61.21% and 38.79% of the total nodes, respectively, with much greater positive interactions (86.02%) than negative interactions (13.98%). Four major modules, namely, modules I, II, IV, and V, were found here, accounting for 43.34% of the nodes. Proteobacteria, Bacteroidota, Ascomycota, and Basidiomycota were the predominant phyla comprising the main modules (Fig. S5). With respect to the subcommunity, compared with specialists, bacterial generalists significantly occupied a greater proportion of important nodes (Fig. 3C; 57.68% vs. 1.24%). Nevertheless, the number of specialists in the fungal community, with 71.29% nodes (including keystone vertices), was approximately four times greater than that in generalists (Fig. 3D). There were much higher percentages of positive correlations among nodes in the two microbial taxa, that is, 74.64% for bacteria and 99.98% for fungi. The modularity indices of both the bacterial and fungal communities exceeded 0.4, indicating that the network had a modular structure and could be divided into several independent functional groups. Three primary modules were classified in both taxa, which consisted mainly of bacterial generalists and fungal specialists (Fig. 3C and D). These modules were principally associated with Proteobacteria (79.54%) and Bacteroidota (28.63%) in bacteria, as well as Ascomycota (48.84%) and Basidiomycota (46.35%) in fungi (Fig. 3C and D). Comparisons of topological features between microbial generalist and specialist taxa revealed that compared with their specialist counterparts, bacterial generalist taxa presented significantly greater numbers of nodes, edges, average degree, and complexity (*P* < 0.01 or 0.001; Fig. 4A). Conversely, in the fungal community, the specialists displayed higher values for these network metrics (*P* < 0.05 or 0.01; Fig. 4A). Furthermore, a robustness test based on natural connectivity was conducted to assess the network invulnerability. The results of the linear regression model revealed that compared with their respective specialist and generalist counterparts, the natural connectivities in both the bacterial generalist and fungal specialist subnetworks exhibited steeper slopes with higher degrees of fit (*P* < 0.05 or 0.001), indicating weakened resistance. These findings suggest that bacterial generalists and fungal specialists play important roles in maintaining the community stability and interactions of the algae-associated microbiome throughout algae growth.

### Evolutionary trends of microbial generalists and specialists

**Fig 5:**
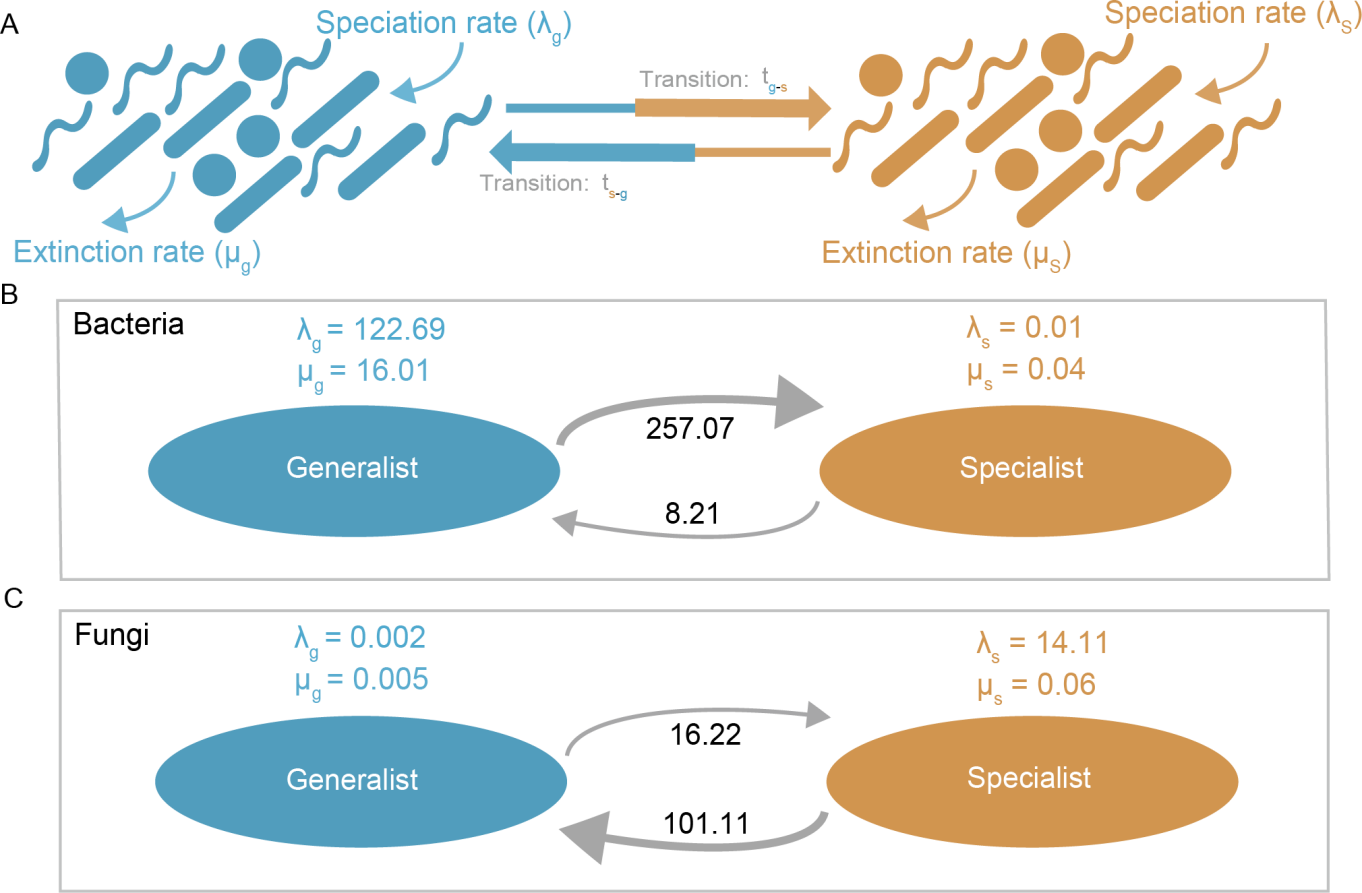
Evolutionary processes of microbial generalists and specialists based on the BiSSE model (1,000 permutations of each parameter). (**A**) Binary-state speciation and extinction model for the evolution of niche generalists and niche specialists. (**B, C**) The variation trend of the per species’ speciation rate, extinction rate, and transition rate for generalists and specialists in bacteria and fungi communities during the growth process.

The average per-species evolutionary rate parameters, including speciation (λ), extinction (μ), and state-transition rates (t), of microbial generalists and specialists in terms of algae growth were estimated through the BiSSE model with 1,000 permutations (Fig. 5). Speciation and transition rates contribute to the increase in microbial diversity, whereas extinction reduces diversity. Bacterial generalist taxa (λ_g_ = 122.69) showed a much higher speciation rate than that of their specialist counterparts (λ_g_ = 0.01) and a greater extinction rate (μ_s_ = 16.01 for generalists; 0.04 for specialists) (Fig. 5B). The rate of transition from generalist to specialist lineages (t_g-s_ = 257.07) was much higher than that of the reverse (t_s-g_ = 8.21). In terms of fungi, completely opposite patterns were observed, with specialist taxa showing higher speciation (λ_g_ = 14.11) and extinction (μ_s_ = 0.06) rates than generalist taxa did (Fig. 5C). Compared with the reverse transition, a more favorable transition from specialist to generalist lineages (t_s-g_ = 101.11) was detected (t_g-s_ = 16.22). Taken together, the above results indicate the important role of bacterial generalists and fungal specialists in regulating the microbial diversity of algae-associated microbes during algae proliferation.

## DISCUSSION

Like microbes inhabiting the rhizosphere and human gut (45, 46), algae-associated microbiota chronically coexist with microalgae, reflecting potentially intimate relationships with the host (17, 18, 24, 47). To date, most studies have focused mainly on meta microbes during HABs (14–16). However, several algae-associated taxa are present within the meta community in natural environments (47). Currently, little attention is still paid to such microbiomes (17, 18). Given the challenges of discerning these species from the whole microbial community in the field, long-term cocultivation between microalgae and microbes may allow us to differentiate them (17, 18, 24, 47). Efforts have been made to elucidate the diversity and community of algae-associated microbes, which helps understand complex algal–microbe interactions (17, 18, 23), especially for HAB-forming species. Nevertheless, in terms of algae-associated microbes with different niches, the contributions and interactions of these microbes along with a harmful algal growth cycle remain underresearched. The present study comprehensively explored the ecological and evolutionary features of generalists and specialists of the algae-associated microbiome during the growth of a toxic HAB dinoflagellate species.

### Discrepant diversity patterns of niche microbes between bacterial and fungal communities

Our results clearly demonstrated that the biodiversity and richness significantly differed between bacterial generalists and specialists (Fig. 1), with much higher values in the former. By contrast, compared with their generalist counterparts, fungal specialists were clearly richer and relatively abundant in biodiversity (Fig. 1). These findings revealed the disparate patterns of niche differentiation between bacterial and fungal taxa. It has been well documented that compounds released by algae impact microbial community structures, as their growth differs in response to these compounds (16, 48–50). In addition, trophic competition often occurs among bacterial and fungal taxa (51). Together with the different nutrient metabolisms between microbial generalists and specialists (10, 52), the niche patterns of microbes described here may be the result of discrepant substance preferences for algal metabolites (e.g., organic acids, amino acids, proteins, and lipids) among niche prokaryotes and microeukaryotes, as well as diverse interactions in the microbiosphere over long-term coculture. Furthermore, despite the comparable biodiversity between the bacterial and fungal communities, algal proliferation led to an increasing trend in the diversity of the former, whereas the opposite phenomenon was observed in the latter (Fig. 1), indicating that more adverse effects were exerted on the fungal community. As mentioned above, differences in nutritional competitive ability may be important (51). In addition, for bacteria, a significantly positive correlation was observed between the diversity of the generalist subcommunity and its whole community (Fig. 1F), whereas that between specialist taxa and the whole consortium differed, indicating that generalists play a more crucial role in maintaining algae-associated bacterial diversity. With respect to fungi, the diversities of both specialists and generalists were positively correlated with that of the overall community (Fig. 1H and K), suggesting that all these factors contribute importantly to fungal diversity dynamics, especially the former. Consequently, community niche breadth was observed to undergo substantial amplification and reduction in bacterial and fungal populations, respectively (Fig. 2B and D), signifying different changing trends in resource availability between the two microbes. Compared with specialists, microbial generalists are generally thought to have greater tolerance to environmental fluctuations (5, 52, 53), which supports the increased and decreased diversities of bacterial generalist and fungal specialists, respectively, but disagrees with the diversity dynamics in niche-generalized fungal taxa (Fig. 1). This may be associated with the much lower richness of fungal generalists or more toxic effects from the studied algae on them, resulting in weak competition with bacterial generalists (Fig. 1G). However, the proliferation of *A. pacificum* profoundly affected the community structures of algae-associated bacteria and fungi, leading to a distinct temporal succession of species composition throughout the process, which is consistent with the findings of other previous studies (15, 25, 54). In terms of niche microbes, compared with generalists, the two types of specialists were more susceptible to HAB influence, as shown by their greater community dissimilarity among growth stages (Fig. 2) and drastic fluctuations in the composition of the top species (Fig. S4). In other words, the composition shift of specialist species occurred at a relatively fixed niche (i.e., a growth stage), whereas the composition turnover of the generalist subcommunity across the growth process was gradual and mirrored the whole community. Not surprisingly, these findings may be attributed to a narrower Levins’ niche breadth in specialist taxa (Fig. S3), which is consistent with other literature-reported results (2, 52). These findings collectively demonstrate that niche differentiation in terms of community composition between different microbial types (i.e., bacteria and fungi) and subcommunities (i.e., specialists and generalists) influences the dynamics of both microbial diversity and community composition under HAB perturbation.

### Community assembly of microbial generalists and specialists is regulated by different ecological processes

Community assembly processes are important drivers of microbial diversity and composition (8, 38). The assembly of microbial communities is generally believed to be governed by two distinct mechanisms, stochastic and deterministic processes, which operate in conjunction with each other (8). Stochastic processes, rooted in the neutral theory, encompass the influences of random events, including birth, death, ecological drift, and probabilistic dispersal. By contrast, deterministic processes, guided by niche theory, pertain to the selective effects of biotic and abiotic factors on biological communities (8, 55). Determining their relative importance in shaping community composition under HABs is helpful for improving the understanding of the mechanisms that regulate microbial distribution patterns (15). In this study, deterministic processes dominated in both bacterial and fungal specialist taxa, whereas stochastic processes played an important role in their generalist community assembly (Fig. 1I and K), which is consistent with a study indicating that if more microbial generalists are present, a stronger stochastic role in microbial community assembly could occur during a diatom bloom process (56). These findings suggest that the algae-associated specialist community was more sensitive to the influence of HAB-induced species proliferation, which is consistent with the abovementioned results (Fig. 2). Furthermore, interestingly, the role of deterministic processes gradually increased in bacterial generalists, and a heightened stochastic effect was observed in their specialist counterparts during the studied process (Fig. 2J and L). However, those in the fungal community exhibited the opposite results. These findings may be ascribed to evolutionary processes between niche generalists and specialists of bacterial and fungal communities, which are important for microbial generation, adaptation, and maintenance (2, 10, 37). Indeed, we observed a higher transition rate from bacterial generalists to their specialists, as well as from fungal specialists to generalist counterparts (Fig. 5), which may have resulted in more deterministic effects on the community assembly of both bacterial generalists and fungal specialists. Despite these findings, the overall patterns of assembly processes in niche generalists and niche specialists did not obviously change (Fig. 2I and K), possibly because of the higher speciation rate of bacterial generalists and fungal specialists (Fig. 5), thus offsetting species losses caused by niche transformation. Taken together, the above results reveal the distinct community assembly processes between microbial generalists and specialists during this process, providing novel insight into how HAB species proliferation affects the algae–bacteria commensal system.

### More stable and complex interaction networks appear in bacterial generalists and fungal specialists

Molecular ecological networks provide valuable insights into biological interactions, such as mutualism, competition, and predation (57). These intricate interdomain relationships, involving prokaryotes and microeukaryotes, as well as intradomain associations within prokaryotes or microeukaryotes, play a vital role in preserving community diversity, stability, and ecosystem functions (37, 53). Here, a substantial abundance of positive intradomain and interdomain interactions was predominantly observed, as opposed to negative interactions (Fig. 3). These findings suggest the existence of more mutualistic relationships within the algae-associated microbial communities, and these positive associations may play a role in preserving biodiversity and sustaining ecosystem functions amidst the influence posed by HABs (58). Furthermore, comparisons of topological features between microbial generalists and specialists were conducted. A much higher percentage of bacterial generalists and fungal specialists participated in interactions in the network (Fig. 3), implying that they are more involved in biological communications during the process. Proteobacteria, Bacteroidota, Ascomycota, and Basidiomycota were the main functional taxa within the microbial generalist and specialist groups, which is consistent with previous studies (16, 52, 59). Even so, further exploration of the specific roles they play still needs to be conducted. Moreover, compared with their niche counterparts, bacterial generalist and fungal specialist communities presented greater complexity, greater proportions of major modules (function areas), greater numbers of nodes and edges, greater average degree, and better robustness (Fig. 4), suggesting that they are more important for maintaining ecosystem stability and function (37, 53). In general, compared with specialists, microbial generalists are characterized by greater resistance to changing conditions (4, 5, 53). Hence, from this perspective, understanding the abovementioned roles of bacterial generalists in the current study is not difficult. However, the role of fungal specialists is typically fulfilled by generalists. There are two possible explanations for this: (i) compared with fungal generalists, those with higher phylogenetic diversity and modularity allow the coexistence of more specialists with different traits (Fig. 1L and 3D), hence gradually replacing the position of generalists during the process. Indeed, the richness of specialists was much greater than that of generalists (Fig. 1); (ii) specialized microbial species could sometimes prioritize the consumption of common resources as a means to evade competition and counterbalance the energetic costs associated with survival via evolutionary transition to generalists to enhance their environmental adaptation for community conservation (2, 10, 52, 53). Certainly, a markedly higher transition rate from fungal specialists to their generalized counterparts was found in the present study (Fig. 5). Collectively, the results provide new insights into the complex diversity of interactions among microbes to better understand how they maintain their community and function under HAB species interference.

### Different evolutionary characteristics of niche microbes between bacterial and fungal taxa and implications

In addition to ecological processes, evolutionary aspects, including speciation, extinction, and transition, are involved in controlling microbial diversity (2, 10, 37). Nevertheless, the fossil record, which serves as a primary source of information regarding the evolutionary processes shaping present-day communities, is largely lacking for microbes (60). Consequently, researchers are compelled to rely on extant sequence data for evolutionary reconstructions (61). BiSSE models offer a means to explore the evolutionary characteristics of extant microbial species (62). The net diversification rate refers to the disparity between the rates of speciation and extinction. Both microbial generalist and specialist taxa could exhibit high rates of diversification, albeit through distinct evolutionary pathways (63). Furthermore, it is proposed that specialists hold higher rates of diversification because they more easily suffer from resource limitation, thus enhancing speciation (64, 65). An alternative perspective posits that generalists possess higher rates of diversification because of their broader niche and distribution, leading to increased allopatric speciation (63). Our work supports these findings; that is, bacteria generalists and fungal specialists had higher speciation rates coupled with lower extinction rates, which resulted in better diversification potential (Fig. 5). Successful speciation is also expected to rely to a greater degree on the population (66). Consistently, a higher richness of bacterial generalists (with broader niches) and fungal specialists (with higher phylogenetic diversity) was found here, possibly increasing the number of survivors and thus indirectly increasing the speciation rate. However, compared with the diversity of bacterial generalists, that of fungal specialists still tended to decrease (Fig. 1K), suggesting that greater diversification may only mitigate rather than halt the decline in diversity under HAB-induced species disturbance. In terms of this speculation, more works still need to be implemented in the future. In addition, despite greater extinction of fungal generalists and bacterial specialists, they experienced continuous replenishment, as their niche counterparts generated relatively stable species pools via higher speciation and maintained a dynamic source–sink relationship with them (Fig. 5), alleviating the “Matthew effect” in the microbial world. Such an evolutionary trajectory implied a “win‒win” scenario among algae-associated generalists and specialists during the process: (i) for bacteria, generalists could alleviate interspecific competitive pressure via transition to specialists (Fig. 1J) to better maintain community and function and enhance adaptation at a specific growth stage; and (ii) for fungi, specialist species achieve niche expansion through transition to generalists for wider distribution across different growth stages to withstand environmental filtration from the process. Despite the disparities in speciation and extinction rates between microbial generalists and specialists, they are evolutionarily linked through transitional processes (Fig. 5). The discovery of this intricate interplay and interdependence between the two has contributed to their coevolution and coadaptation during the process, aligning with a fundamental tenet of the Red Queen theory (67). Hence, the equilibrium of evolutionary dynamics between algae-associated specialists and generalists in this process may be driven by biological factors.

### Conclusion

For the first time, the ecological and evolutionary processes of free-living generalists and specialists within algae-associated bacterial and fungal communities during the growth cycle of a toxic bloom-forming dinoflagellate were comprehensively investigated. There are four main novel findings. (i) Significant differences were observed in the microbial niche patterns between the bacterial and fungal communities. Specifically, bacterial generalists and fungal specialists were predominant in the microbial community, with higher diversity and richness. (ii) Microbial specialists exhibited heightened susceptibility to disturbances arising from algal proliferation, as evidenced by increased community dissimilarity and assembly processes dominated by deterministic factors. (iii) Bacterial generalists and fungal specialists exhibited an ecological network that was not only more intricate but also more resilient and stable, and acted as the main participants in interspecies interactions. (iv) Bacterial generalist and fungal specialist taxa presented higher diversification rates and a better ability to transition into their niche counterparts, contributing to preventing diversity imbalance. Taken together, these findings highlight the pivotal roles of bacterial generalists and fungal specialists in sustaining microbial community stability and biodiversity throughout the growth cycle of HAB species. The findings of the present study expand our knowledge of the ecological significance of microbial generalists and specialists within the algae-associated microbiome during the growth process of HAB-forming species, establish a vital connection between laboratory and field data, and offer crucial theoretical guidance and empirical data support for the management of toxic blooms.

## Data Availability

The raw sequence reads were deposited in the NCBI Sequence Read Archive (SRA) database under accession numbers PRJNA1192050 for bacteria and PRJNA1192698 for fungi.

## SUPPLEMENTAL MATERIAL

Supplemental figures aem.01359-25-s0001.docx Figures S1 to S5.

Table S1 aem.01359-25-s0002.xlsx Community composition of microbial generalists and specialists at genus level.

ASM does not own the copyrights to Supplemental Material that may be linked to, or accessed through, an article. The authors have granted ASM a non-exclusive, world-wide license to publish the Supplemental Material files. Please contact the corresponding author directly for reuse.
