# Supplementary material for: Bacterial generalists and fungal specialists play important roles in maintaining community stability and regulating microbial diversity of the algae-associated microbiome throughout the growth cycle of Alexandrium pacificum
Source: Appl Environ Microbiol. 2025 Sep 22;91(10):e01359-25. doi: 10.1128/aem.01359-25 (PMC12542695; doi:10.1128/aem.01359-25)
Supplement: Supplemental figures — Figures S1 to S5. [file aem.01359-25-s0001.docx]

**
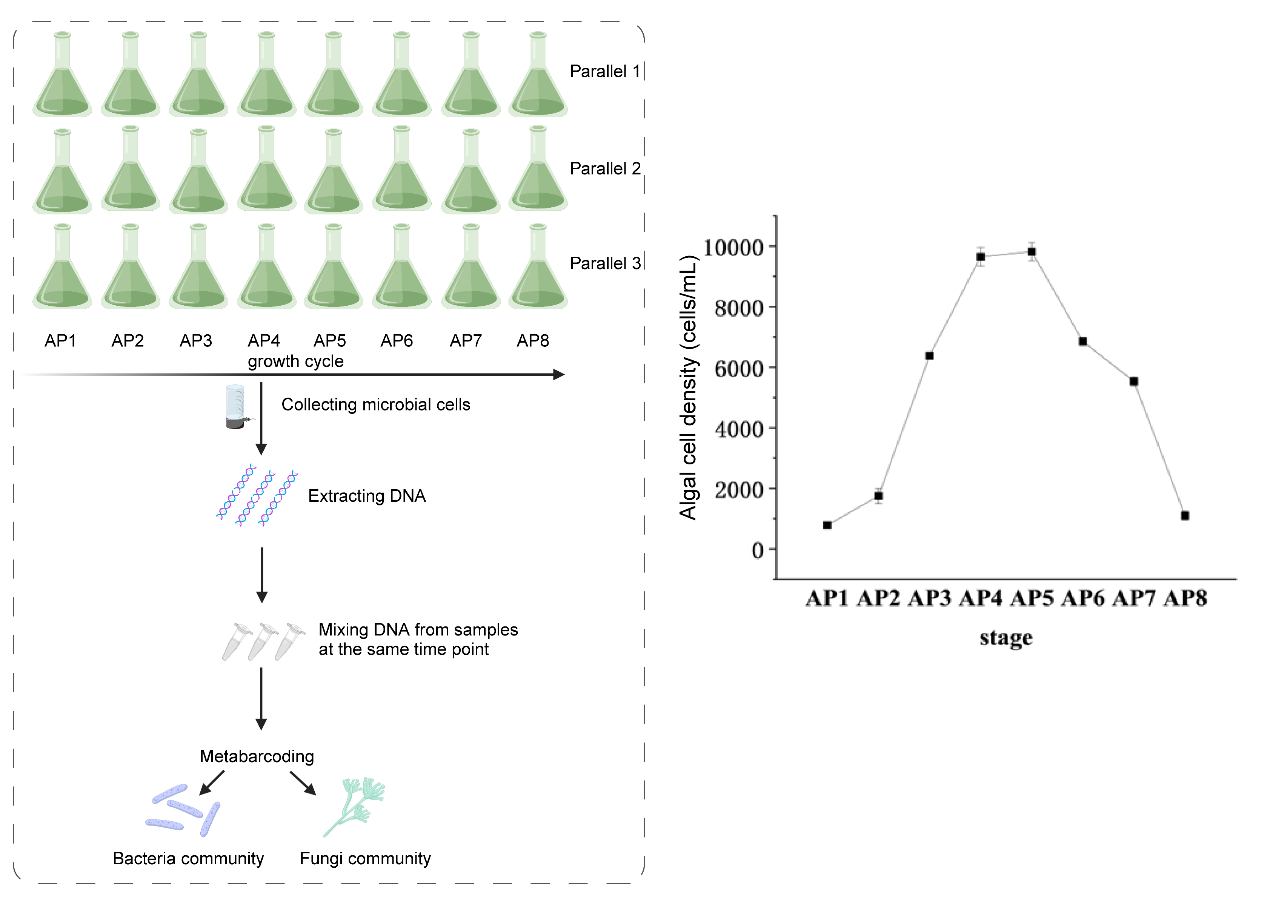
**

**Supplementary Figure 1.** Sample processing and cell counts for *A. pacificum* at different sampling times.


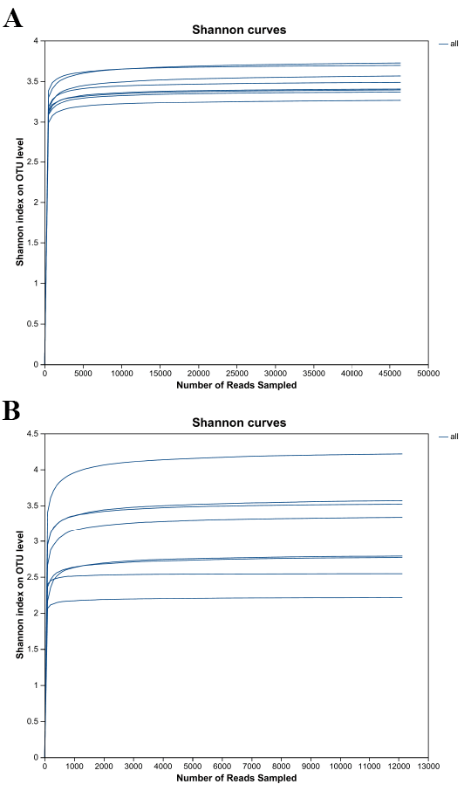


**Supplementary Figure 2.** Rarefaction curve. (A) bacterial community; (B) fungal community.


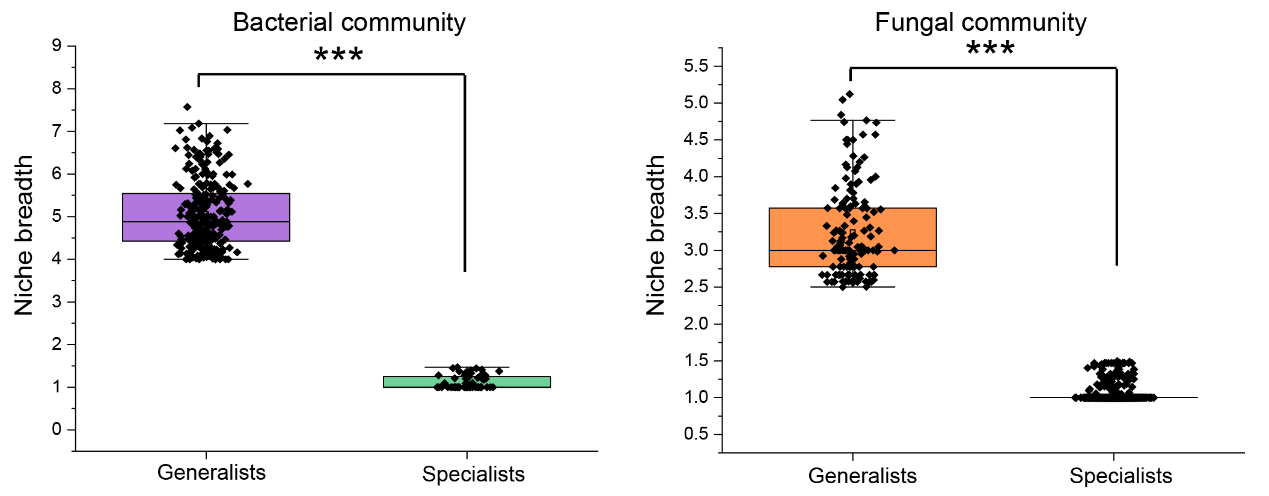


**Supplementary Figure 3.** Niche breadth of microbial specialists and generalists. ***, P < 0.001.


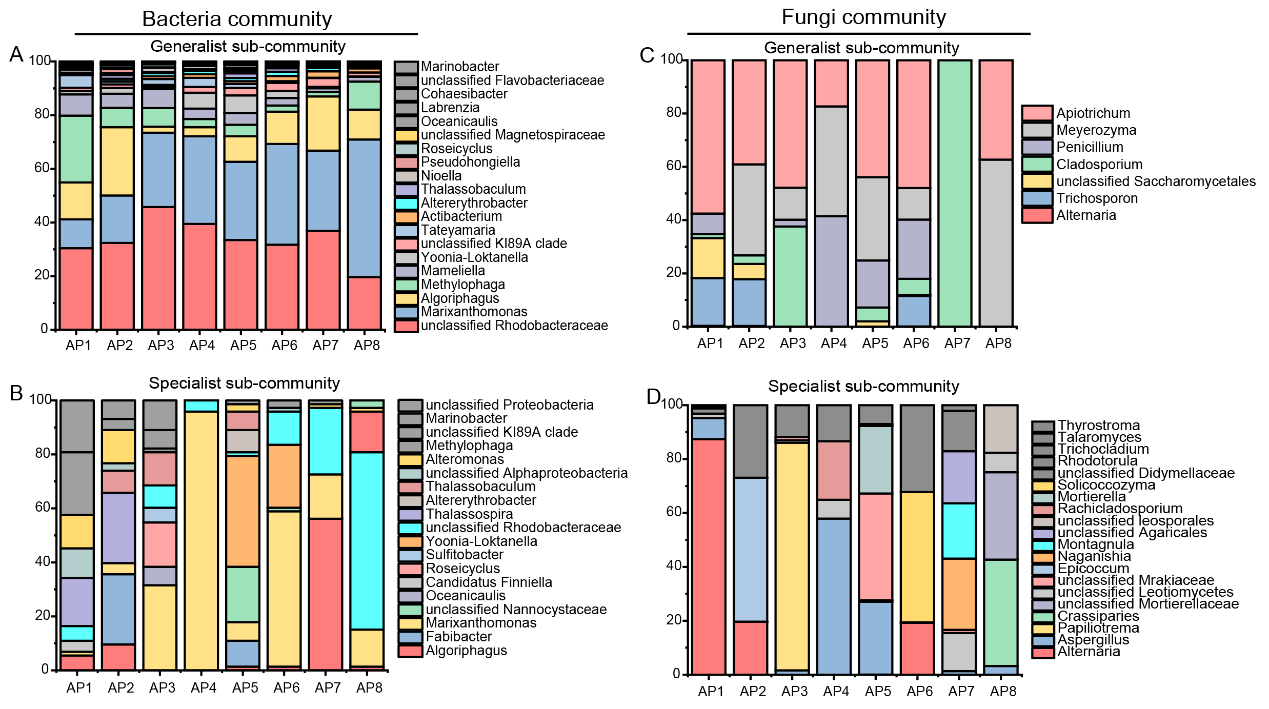


**Supplementary Figure 4.** Change of relative abundance of top 20 genera of bacteria and fungi.


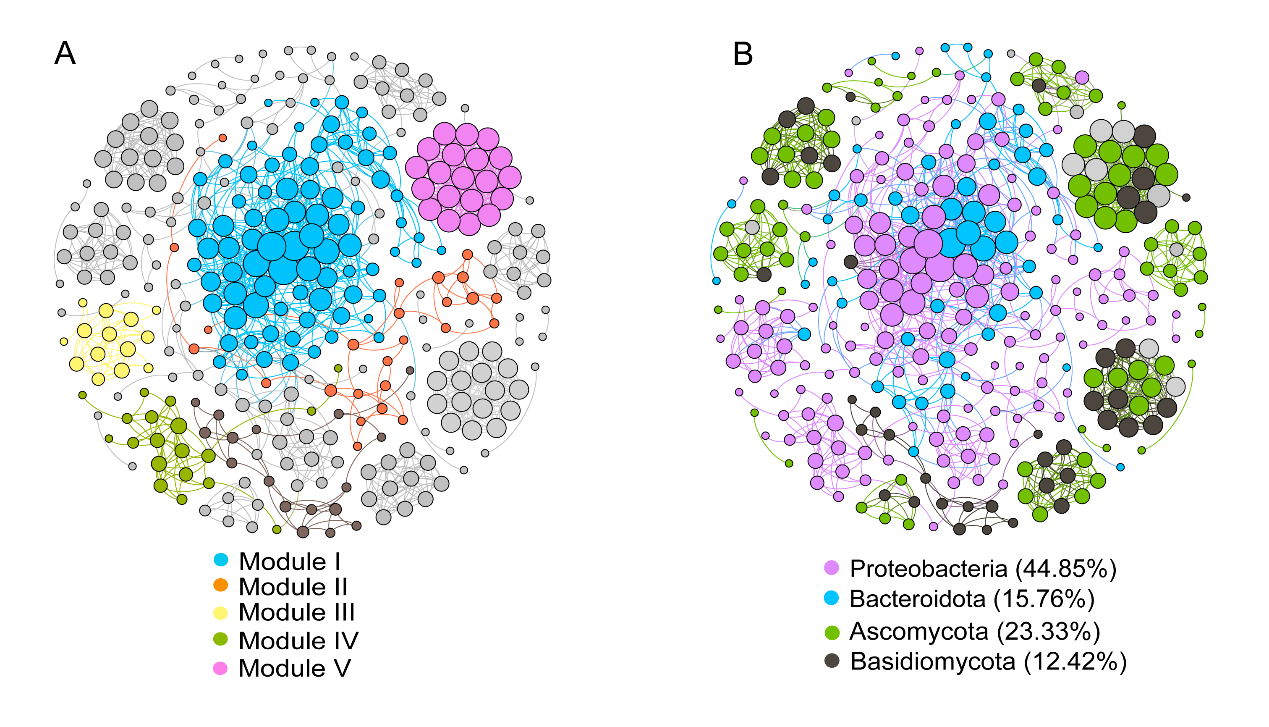


**Supplementary Figure 5.** Co-occurrence networks of whole-community (bacteria + fungi). (A) Distribution pattern of modules; (B) Major species at phylum level of modules.
